# Pediatric hypertension screening: a disparity between guidelines and practice

**DOI:** 10.1097/MS9.0000000000005125

**Published:** 2026-05-01

**Authors:** Zia Ullah, Muskan Asghar Qureshi, Shahzadi Barjis Rashid, Zain Ul Abedeen, Abad Ur Rehman

**Affiliations:** a Department of Medicine, Saidu Medical College, Swat, Pakistan; b Department of Medicine, Liaquat University of Medical Health and Sciences, Jamshoro, Pakistan; c Department of Medicine, Bahria University of Medical and dental College, Karachi, Pakistan; d Department of Medicine, Spinghar University (Kabul campus), Kabul, Afghanistan

*Dear Editor,*

Pediatric hypertension (PH) is one of the increasingly prevalent and often overlooked public health issues. It is recognized as a persistent rise in blood pressure at or above the 95th percentile for age, sex, and height, on a minimum of three distinct clinical encounters. Diagnosing PH in children is complex due to the need for percentile-based evaluation, as compared to the fixed thresholds used in adults**^[^1^]^**.

Recent data report a higher rate of occurrence of PH than previously reported. A 2026 meta-analysis, published in *The Lancet Child & Adolescent Health*, reports that the true rate of PH, as determined by out-of-office monitor incidence, is 6.67%. However, the reported worldwide occurrence of hypertension based on repeated in-office readings is 4.28%**^[^2^]^**. This distinctiveness implies that using only conventional in-office measurements could result in insufficient recognition, especially of conditions like masked hypertension. Such underrecognition, combined with the long-term outcomes of PH, which are severe, warrants the need for routine screening and prompt management. A population-based cohort from 2024 showed that children presenting with hypertension are at a higher risk of adverse cardiac events (myocardial infarction, stroke, etc.) in their adult age compared to their normotensive peers. These adverse cardiac events might include myocardial infarction, arrhythmias, and stroke, among others**^[^3^]^**.

Despite a clear indication for screening, still there is a significant implementation gap between the 2017 guidelines suggested by the American Academy of Pediatrics and routine clinical practice**^[^4^]^**. A question arises as to why diagnostic inertia persists despite the presence of such widely accepted guidelines. Therefore, we suggest the decisive need to go beyond these repetitive guidelines and apply a tailored approach that challenges the existing obstacles in the detection of PH. This subject was specifically examined in a recent qualitative study, which highlighted various important modifiable obstacles, all identified by clinicians. These included the shortage of defined clinical pathways, insufficient staff confidence and training, as well as electronic medical records that do not facilitate blood pressure trend analysis over time**^[^5^]^**. Effective screening and follow-up are severely impeded by these system-level hindrances, as well as disconnected communication with subspecialists.

These issues are particularly relevant in countries like Turkey, where rates of childhood obesity are reported to be rising significantly. As obesity is a major contributor to hypertension, significant clinical attention is required in this regard. According to a study from a Turkish Nephrology Center published in 2025, 60.3% of the 189 children who participated in the study, when examined under ambulatory blood pressure monitoring, had hypertension, and an additional 9.5% had borderline blood pressure**^[^6^]^**. Crucially, compared to ideal blood pressure controls, patients presenting with high blood pressure showed substantially reduced nocturnal dipping, leading to an increased risk of cardiovascular disorders^[^6^]^. These reported data highlight the need for thorough screening and follow-up visits. It also suggests that, as PH might be more common in such demographics, it could lead to early target-organ damage in these children.

Accordingly, we suggest that raising awareness alone is not enough to overcome the evidence–practice gap. Targeted health system interventions are required, such as developing a structured referral system involving subspecialists, implementing competency-based training for all clinical personnel based on appropriate measuring techniques, and improving electronic health records to identify concerning readings based on percentiles**^[^5^]^**. By tackling the highlighted obstacles and executing clinical guidelines in routine practice, we can eventually decrease the long-term cardiovascular burden associated with juvenile hypertension. By directly challenging such systemic hurdles in execution, we can go beyond just guideline distribution to achieve effective implementation. Such proactive strategies are required to tackle PH and limit its long-term cardiovascular adverse effects.

## Data Availability

All the data used in the study are available online and can be accessed through the reference list.

## Generative AI

This editorial is in line with the TITAN guidelines for the use of generative AI in medical research. A thoroughly completed TITAN checklist has been provided alongside the manuscript.

## References

[1] Falkner B Sadowski RH. Hypertension in children and adolescents. Am J Hypertens 1995;8. doi:10.1016/0895-7061(95)00308-8 8845092

[2] Zhou J Shan S Wu J. Global prevalence of hypertension among children and adolescents aged 19 years or younger: an updated systematic review and meta-analysis. Lancet Child Adolesc Health 2026;10:11–21. 41240955 10.1016/S2352-4642(25)00281-0

[3] Robinson CH Hussain J Jeyakumar N. Long-term cardiovascular outcomes in children and adolescents with hypertension. JAMA Pediatr 2024;178:688–98. 38709137 10.1001/jamapediatrics.2024.1543 PMC11217870

[4] Flynn JT Kaelber DC Baker-Smith CM. Clinical practice guideline for screening and management of high blood pressure in children and adolescents. Pediatrics 2017;140. doi:10.1542/peds.2017-1904 28827377

[5] Zaidi AH Sood E De Ferranti S. Clinician perceptions of barriers and strategies to improve pediatric hypertension detection. JAMA Netw Open 2026;9:e2560542–e2560542. 41758513 10.1001/jamanetworkopen.2025.60542 PMC12949442

[6] Aytac MB Doğan K Ergül ŞA. Ambulatory blood pressure monitoring in children: single center experience. Türkiye Çocuk Hastaliklari Dergisi 2025;19:43–48.

